# Lethal pulmonary embolism in a pregnant woman with severe acute respiratory syndrome coronavirus-2 receiving prophylactic anticoagulation: a case report

**DOI:** 10.1186/s13256-023-04180-w

**Published:** 2023-11-02

**Authors:** Yuliya V. Perfilyeva, Saule B. Maukayeva, Yerbol M. Smail, Andrey M. Dmitrovskiy, Yekaterina O. Ostapchuk, Andrey V. Zhigailov, Anna S. Nizkorodova, Zhanna A. Berdygulova, Dinara A. Naizabayeva, Anastassiya V. Perfilyeva, Elina R. Maltseva, Kulyan Zh. Kamytbekova, Yuriy A. Skiba

**Affiliations:** 1 Almaty Branch of the National Center for Biotechnology, Central Reference Laboratory, 14 Zhahanger St., Almaty, 050054 Kazakhstan; 2 Semey Medical University, 103 Abay Kunanbayev St., Semey, 071400 Kazakhstan; 3 M. Aikimbayev’s National Scientific Center for Especially Dangerous Infections, 14 Zhahanger St., Almaty, 050054 Kazakhstan; 4 Al-Farabi Kazakh National University, 71 Al-Farabi Avenue, Almaty, 050040 Kazakhstan; 5 Institute of Genetics and Cytology, 93 Al-Farabi Avenue, Almaty, 050060 Kazakhstan; 6 South-Kazakhstan Medical Academy, 1 Al-Farabi Avenue, Shymkent, 16001 Kazakhstan

**Keywords:** COVID-19, Pregnancy, Pulmonary Artery Embolism, Lethal Outcome

## Abstract

**Background:**

A limited number of studies have described thrombotic complications in pregnant women with COVID-19. Here we report on fatal pulmonary embolism in a pregnant woman with laboratory confirmed SARS-CoV-2 infection.

**Case presentation:**

A 28-year-old Kazakh woman was hospitalized with muscle pain, dry cough and a temperature of 37.5 °C at the 29th week of gestation. Upon admission, a blood test demonstrated elevated neutrophil-to-lymphocyte ratio, decreased levels of erythrocytes and hemoglobin, as well as prolonged prothrombin and activated partial thromboplastin time. Within 14 days of admission, she experienced respiratory distress and underwent transfer to the intensive care unit, intubation and a cesarean section. The patient received intravenous antibiotics, antiviral medications, systemic corticosteroids and dual anticoagulation with aspirin and enoxaparin. Death outcome was reported on day 18 of illness despite aggressive supportive care. Histological analysis demonstrated that obstruction of the main pulmonary arthery and disseminated intravascular coagulation were the causes of death.

**Conclusions:**

This case demonstrates that in the management of pregnancy and childbirth in patients with suspected or confirmed COVID-19 infection, special attention should be paid to coagulation system parameters and timely appropriate prophylaxis of thromboembolic complications, which has yet to be determined.

## Background

First registered in December, 2019 in Wuhan, Hubei Province, China, coronavirus disease 2019 (COVID-19) spread all over the world affecting more than 766 million people, as of May 22, 2023 [1]. It is caused by severe acute respiratory syndrome coronavirus-2 (SARS-CoV-2), which primarily affects the respiratory system [2]. According to the data of Johns Hopkins University School of Medicine, the case fatality rate of COVID-19 ranges between 0.2% and 9.2% depending on the country [3]. Older people and people with underlying medical conditions are attributed to the groups at increased risk for severe illness from COVID-19. The Centers for Disease Control and Prevention (CDC) report that pregnant women are also at increased risk for severe illness from COVID-19 [4].

Information available so far suggests that COVID-19 during pregnancy rarely affects fetal and neonatal mortality [5–7]. The information on COVID-19-related maternal mortality continues to be updated. Initial reports from China did not indicate fatal outcomes among pregnant women with COVID-19 [8]. A systemic review of 230 cases found that the mortality of pregnant women with COVID-19 was lower than that of overall COVID-19 patients and similar to the average maternal mortality rate worldwide (1 in 180 cases) [7]. A number of national population-based cohort studies demonstrated that among women with SARS-CoV-2 infection, pregnancy was associated with increased rates of intensive care unit (ICU) admission and the need for mechanical ventilation but not death [9–11]. In contrast, as of November, 2020, an updated report from CDC on 409,462 women showed that COVID-19 was associated with a higher risk of death among pregnant women when compared to non-pregnant women [12]. Similar results were obtained in an anlysis of data from more than 11,400 women with confirmed or suspected COVID-19 [13]. Alarming data on high fatality rates among COVID-19-infected pregnant women have been reported in some countries [14, 15]. According to the official data, a more than twofold increase in the maternal mortality rate was observed in Kazakhstan in 2020 during the initial wave of COVID-19 (36.5 deaths per 100,000 live births as compared to 13.7 deaths per 100,000 live births in 2019) [16]. Of course, some of this mortality can be attributed to the insufficient quality of prenatal and antenatal care, as well as the lack of hospital resources (medical staff, medications) to manage critical situations due to the pandemic. Nevertheless, the statistics might also reflect the direct impact of the disease on pregnancy. Therefore, clinicians are encouraged to publish case reports providing information on the disease course, treatment and outcomes of SARS-CoV-2 infection in pregnancy with the final aim to prevent other deaths.

In this study, we report on the disease progression, delivery and lethal outcome in a 28-year-old Kazakh woman with no preexisting comorbidities admitted to a hospital at the 29th week of pregnancy with PCR-confirmed COVID-19, and then discuss the case.

## Objectivs and study design

We retrospectively reviewed medical records of a pregnant woman diagnosed with COVID-19. The woman spent the entire diagnosis and treatment process at the Rudny City Hospital, Kazakhstan, under the care of medical staff. This study was reviewed and approved by the Ethics Committee of the National Center for Biotechnology (approval #4 issued on September 8, 2020).

## Case presentation

On June 23, 2020, the patient, a 28-year-old Kazakh woman at the 29th week of pregnancy, Gravida 3 Para 3, developed a dry cough, followed by an elevated temperature of 37.5 °C and muscle pain on the next day. Antipyretics had no effect, and the cough worsened.

On day 5 of illness, she was admitted to the hospital with a diagnosis of acute viral respiratory infection, presumably COVID-19. On examination, she looked pale, had shortness of breath at rest that worsened with mild exertion, and cough with mucopurulent sputum that was difficult to clear. The pharynx was moderately hyperemic. The patient had a temperature of 37.1 °C, a pulse of 89/minute, a blood pressure of 100/60 mmHg, 18 breaths per minute at rest, a normal chest auscultation, and an oxygen saturation (SpO_2_) of 99% on room air. Body mass index, calculated as weight in kilograms divided by height in meters squared, was 18.9. The liver and spleen were not enlarged. No swelling in the lower extremities were observed. Bimanual examination showed soft uterus enlarged to the size of 29 weeks of gestation and a normal longitudinal position of the fetus. Neurologic examination revealed no abnormalities, the patient was conscious and able to make contacts without difficulty. She had a history of mild chronic iron-deficiency anemia and positive ELISA-test results for IgG to cytomegalovirus and herpes simplex virus, but no other significant medical history. The patient was employed in a nursing home. She lived with her husband and three children. The patient did not smoke or consume alcohol and denied any family history of vascular or other chronic problems. No problems were noted during regular prenatal examinations, so she was not receiving any medications prior hospitalization.

Upon admission, a SARS-CoV-2 PCR test of a nasopharyngeal swab confirmed the diagnosis COVID-19. According to the protocols established in Kazakhstan at that time for the treatment of COVID-19 in pregnant women, antimicrobal treatment (1.0 g ceftriaxone intramuscularly twice daily for 3 days, then 1.0 g ertapenem intravenously once daily for 11 days and 400 mg moxifloxacin intravenously twice daily for 11 days, 100 ml fluconazole 2 mg/ml intravenously once) was initiated.

On day 7 after onset of symptoms, the patient's condition worsened with a respiratory rate of 27–31 breaths per minute at rest, tachycardia up to 120/min and 93% SpO_2_. Performed chest radiography showed 2-sided total pneumonia. The patient was transferred to the ICU, where non-invasive ventilation was initiated. The laboratory findings included elevated neutrophil-to-lymphocyte ratio and erythrocyte sedimentation rate, decreased erythrocytes and hemoglobin, as well as prolonged prothrombin (PT) and activated partial thromboplastin (APTT) time (Table 1). The patient received antiretroviral drugs (200 mg lopinavir and 50 mg ritonavir orally twice daily for 9 days), glucocorticosteroids (8 mg dexamethasone intravenously twice daily for 11 days) and anticoagulants (100 mg aspirin orally once daily for 11 days and 40 mg enoxaparin subcutaneously once daily for 11 days).

**Table 1:** Laboratory findings of the patient

Marker (reference range)	Day of illness							
	5th	7th	8th	10th	11th	13th	17th	18th
Blood analysis								
Leukocyte count (4.5–9.5 × 10^9^/L)	–	5.8	8.1	8.1	11.3	–	5.6	5.6
Neutrophil (1.8–6.3 × 10^9^/L (50–70%))	–	5.2 (89%)	7.1 (87%)	7.1 (87%)	10.6 (94%)	–	–	3.7 (66%)
Lymphocyte (1.1–3.2 × 10^9^/L (18–40%))	–	0.5 (9%)	0.9 (11%)	0.9 (11%)	0.5 (4%)	–	–	2.2 (31%)
Neutrophil: Lymphocyte ratio	–	10.4	7.9	7.9	21.2	–	–	2.1
Monocytes (0.2–1.0 × 10^9^/L (2–8%))	–	0.06 (1%)	0.07 (1%)	0.06 (1%)	0.06 (0.5%)	–	–	0.12 (2%)
Eosinophils (0.02–0.5 × 10^9^/L (0–5%))	–	0.05 (1%)	0.06 (0.5%)	0.06 (0.5%)	0.05 (0.5%)	–	–	0.06 (1%)
Platelets (125–350 × 10^9^/L)	–	200	230	200	130	–	–	–
Erythrocytes (3.8–5.8 × 10^12^/L)	–	2.95	3.1	2.85	2.7	–	2.98	2.98
Hemoglobin (115–150 g/L)	–	85	80	80	75	–	89	89
Alanine transaminase (7–40 U/L)	–	–	9.1	7.4	7.4	69.5	37.2	37.2
Aspartate transaminase (13–35 U/L)	–	–	25.6	21.0	21.04	80.4	34	34
Creatinine (45–90 mcmol/L)	–	54	60	57	–	67	119	119
Urea (2.1 to 8.5 mmol/L)	–	–	2.4	3.2	–	5.8	10.9	10.9
Total bilirubin (1.71–20.5 mcmol/L)	–	–	11.2	15.5	–	11.3	21	21
Total protein (64–83 g/L)	–	73	56.5	55	55	55	60	60
D-dimer (0.0–0.5 µg/mL)	–	0.6	0.9	0.9	–	–	–	–
Erythrocyte sedimentation rate (15–25 mm/h)	–	40	25	25	25	–	15	15
Blood gas pH (7.35–7.45)	–	7.39	7.3	7.3	7.3	7.28	7.37	7.37
Activated partial thromboplastin time (23–30 s)	–	45	45	36	36	29	60	60
Fibrinogen (1.5–3 g/L)	–	2.2	2.2	5.5	5.55	1.99	3.1	3.1
Prothrombin time (11–13.5 s)	–	17	17	17	17	-	20	20
INR (0.8–1.2)	–	1.12	1.12	1.2	1.2	1.1	1.4	1.4
Urinalysis	–							
Color (yellow)	–	Yellow	Yellow	–	Yellow	Yellow	Yellow	Yellow
Appearance (clear)	–	Clear	Clear	–	Clear	– Clear	-	Not clear
Urinary specific gravity (1.002–1.035)	–	1.107	1.007	–	1.022	1.017	1.018	1.018
pH (4.5 to 8)	–	–	5	–	5	5	5	5
Proteins (les or = 150 mg/l)	–	69	69	–	183	74	70	175
Erythrocytes (0–5 cells/high-power field)	–	–	–	–	12	15	7	9
Leukocytes (0–5 cells/high-power field	–	5	–	–	2	5	8	–
Epithelial cells (0–20 squamous epithelial cells/high-power field)	–	3	3	–	1	5	12	12
SARS-CoV-2 PCR	Positive	–	–	–	–	–	–	–
Blood culture test	–	–	Negative	–	–	–	–	–
Treatment	Antimicrobal and detoxification therapy	Ritonavir, glucocorticosteroids, anticoagulants; connected to ventilators	Endotracheal intubation	20% albumin	-	Prone position	Prolonged ventilations with propofol and atracurium,	–
Outcome	Hospitalization	–	–	Emergency delivery through a cesarean section	–	–	–	Death

*INR* International Normalised Ratio, *PCR* Polymerase Chain Reaction, *SARS-CoV-2* Severe Acute Respiratory Syndrome Coronavirus 2

**Fig.1:**
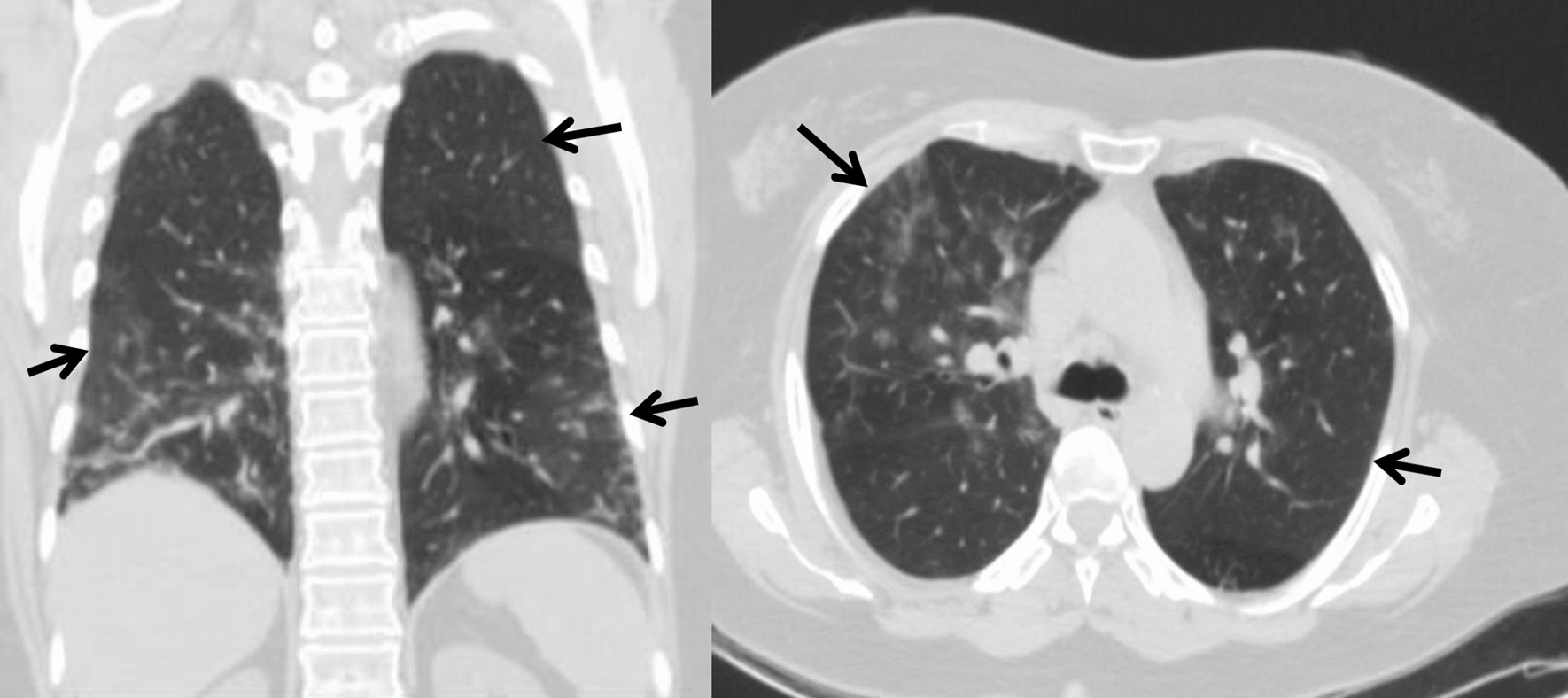
Nonenhanced chest computed tomography images of the patient on day 8 of illness. The computed tomography scans showed bilateral, patchy, ground-glass opacities (arrows)

On day 8 of illness, the patient’s respiratory status acutely deteriorated. The oxygen saturation dropped to 85% SpO_2_ prompting further investigation with computed tomography (CT). A CT scan showed multiple ground-glass opacities located in the subpleural and middle zones of multiple lobes of both lungs (Fig. 1). A total of 84% of both lungs was affected. No pathological changes in the large vessels of the upper mediastinum, heart, pericardium or esophagus were found. Lymph nodes (paratracheal and aortopulmonary) were not enlarged. The patient was intubated. Puncture and catheterization of the right jugular vein were performed.

On day 10, the patient complained of weakness, increased fetal activity and recurrent frequent moderate pain in the lower abdomen. A cough with difficulty separating mucopurulent sputum was noticed. Given the threatening condition for the fetus, an emergency delivery through a cesarean section was performed. A 1,400 g female newborn was delivered at the 30th week of gestation. SARS-CoV2 PCR test was performed with a nasopharyngeal swab and the result was negative. After the surgery, the patient was transported back to the ICU for continued ventilation and supportive care. A standard of care included 200 ml normal saline once, 100 ml of 20% albumin intravenously once, 5% ketamine intramuscularly once daily for 3 days, 100 mg ketoprofen intramuscularly twice daily for 6 days, arduan once, 25 mg atracurium besilate intravenously once daily for 5 days, 5 IU oxytocin intramuscularly once daily for 4 days, 5 mg metoclopramide intravenously once.

On day 13 of illness, arterial blood gasses demonstrated worsening metabolic acidosis. The laboratory analysis showed increased alanine transaminase and aspartate transaminase together with a decreased total protein level suggesting multiple organ failure (Table 1). The patient was placed in the prone position.

On day 17 of illness, the patient's condition remained critical. Prolonged mechanical ventilation with sedation by propofol was initiated (500 mg propofol twice daily). Monitoring of blood pressure showed fluctuations from 90/60 mmHg to 100/65 mmHg and a pulse of 89/min.

On day 18, the patient experienced acute hypotension (blood pressure of 70/45 mmHg), tachycardia (pulse of 167/min) and a fever of 40.0 °C. Later that day, despite maximal ventilator support a lethal outcome was reported. The clinical details of the patient are shown in Table 1 and the chest CT image is shown in Fig. 1.

Performed autopsy demonstrated complete thrombotic occlusion of the main pulmonary arthery lumen. Widespread hemorrhage and vascular thrombosis in the lungs, kidneys, uterus, heart and liver were detected. Lung histological findings included pulmonary atelectasis, exudate with erythrocytes, fibrin filaments in the lumen of the alveoli, eosinophilic deposits on the walls of the alveoli, hyalinosis, and blood clots in the vessels.

After rehabilitation, the newborn was released to the care of his father in satisfactory condition. Subsequently, he developed normally.

## Discussion

Here, we present the fatal case of a 28-year-old female patient Gravida 3 Para 3 at 29-week gestation, with a normal body mass index, and no vascular history, who was hospitalized in 2020 with COVID-19. Despite treatment with prophylactic doses of enoxaparin, she died 8 days after cesarean section from pulmonary embolism and disseminated intravascular coagulation. This case represents one of the few available reports of maternal death due to thromboembolic disorders despite thromboprophylaxis.

It is now clear that SARS-CoV-2 elicits systemic inflammation, which results in an imbalance between procoagulant and anticoagulant homeostatic mechanisms leading to thromboembolic disorders [17]. The cumulative rate of thromboembolic events in COVID-19 patients reaches 21%, rising from 7% of patients admitted to the general ward to 28% of patients in the ICU [18]. We believe that the hypercoagulability associated with pregnancy may increase the risk for thromboembolic disorders associated with COVID-19 even more in pregnant women. Large-scale epidemiological studies are needed to confirm or deny this pressing statement. The aim of this study was to demonstrate an urgent need for development of a standardized protocol for thrombosis monitoring and thromboprophylaxis in pregnant women with COVID-19.

Our patient was a 28-year-old pregnant woman who presented with a cough and muscle aches, which alongside with fever have been earlier found to be the most frequently reported symptoms of COVID-19 in both pregnant and non-pregnant women [13]. No serious preexisting comorbidities putting the patient above baseline population risk (gestational diabetes mellitus and subclinical hypothyroidism, hypertension, obesity, cardiovascular diseases, asthma or renal diseases) or previous thrombotic complications have been reported in the patient's history. A 30-week newborn delivered through a cesarean section had normal birth weight and a negative COVID-19 status. Vertical transmission is rarely reported in COVID-19 patients [7] and was not the case in this study.

Laboratory examination of coagulation indicators upon patient’s admission demonstrated slightly elevated D-dimer levels, prolonged PT and APTT, alongside with fibrinogen levels and platelet counts within the normal range. Given the disorder of three coagulation indicators, therapy with prophylactic doses of low molecular weight heparin (LMWH) and aspirin was initiated. However, despite the young age, negative history for thrombosis and adequate antithrombotic prophylaxis with both anticoagulant and antiplatelet drugs, on the eighth day after cesarean delivery she developed a large pulmonary artery embolus and disseminated intravascular coagulation, which led to her death.

Similarly to our case, a recently published case report presented a COVID-19-positive 29-year old women at the 38th week of pregnancy who developed a pulmonary embolism after cesarean delivery despite prophylaxis [19]. Antithrombotic treatment with intermediate doses of LMWH (enoxaparin subcutaneously 60 mg daily) was started in view of her pregnancy and an elevated D-dimer level (1.50 µg/mL). However, on the second day after cesarean delivery the patient was diagnosed with pulmonary thromboembolism in the right lower lobar pulmonary artery. Both cases strongly demonstrate that high degree of clinical suspicion of venous thromboembolism should be adopted for pregnant patients even if they receive antithrombotic prophylaxis.

Lessons to be learned from the case study: D-dimer plays a key role in thromboembolism events [20]. It has been shown that D-dimer levels greater than 1 μg/mL upon admission predict poor prognosis in nonpregnant patients with COVID-19 [21]. One recent study found a sensitivity of 85.0% and specificity of 88.5% for diagnosing VTE in patients with D-dimer levels greater than 1.5 μg/mL [22]. However, D-dimer is difficult to interpret in pregnancy. It has been demonstrated that D-dimer levels were above the conventional cut-off point (0.5 µg/mL) in 99% of pregnant women in the third trimester [23]. In our patient, the levels of D-dimer were shown to be slightly elevated upon admission, suggesting that pulmonary embolism occurred later. Unfortunately, the test was not performed in the later period of hospitalization, which did not let the physicians fully address hemostatic changes after the cesarean section and augment anticoagulation. D-dimer alongside with other coagulation markers have to be vigorously monitored in pregnant women with COVID-19 on a daily basis especially after cesarean delivery due to coexisting prothrombotic conditions. Increased D-dimer levels may prompt pulmonary angiography examination and intensification of anticoagulation. The use of intermediate therapeutic doses of LMWH should be considered on an individual basis in such patients.

The study also demonstrates that in agreement with recommendations of the International Society on Thrombosis and Haemostasis Subcommittee for Women's Health Issues in Thrombosis and Hemostasis [24], prolonged PT and APTT at any stage of hospitalization should not be considered as a contraindication for thromboprophylaxis in pregnant women, but should prompt further evaluation of other coagulation markers.

Another factor to consider is the use of corticosteroids in pregnant women. It has been shown that mortality at 28 days was significantly lower in patients with COVID-19 who received dexamethasone than in those who received standard treatment alone [25]. Regarding the dose of dexamethasone prescribed to the patient in this study (8 mg twice daily), a lower incidence of adverse events was observed in patients with COVID-19 who received 8 mg once daily than in patients who received higher doses of dexamethasone [26]. On the other hand, the beneficial effects of glucocorticoid treatment received by the patient may have been counteracted by adverse effects on coagulation and fibrinolysis. A number of studies have identified glucocorticoid as a risk factor for venous thromboembolism [27–29]. It is difficult to evaluate the risk–benefit of glucocorticoid treatment in this particular case, especially since there are no adequate and well-controlled studies of corticosteroid treatment in pregnant women. Nevertheless, special caution should be exercised when prescribing corticosteroids in preganat women, especially in those with impaired coagulation parameters.

## Conclusion

The risk of thromboembolic events associated with pregnancy is approximately ten times higher than outside of pregnancy. Cesarean delivery is another risk factor for development of venous thromboembolism. On the other hand, it has been shown that COVID-19 is also associated with coagulation dysfunctions. Thus, pregnant women with COVID-19 have at least two risk factors, and cesarean delivery is the third risk factor for developing thromboembolic complications. Therefore, management of pregnancy and childbirth in the presence of suspected or confirmed COVID-19 infection requires a special attention to the monitoring of coagulation markers and timely appropriate prophylaxis of thromboembolic complications, especially when deciding on a cesarean section.

## Data Availability

Not applicable.

## Abbreviations

COVID-19: Coronavirus disease 2019
SARS-CoV-2: Severe acute respiratory syndrome coronavirus-2
CDC: The Centers for Disease Control and Prevention
ICU: Intensive care unit
PCR: Polymerase chain reaction
ELISA: Enzyme-linked immunosorbent assay
PT: Prothrombin time
APTT: Activated partial thromboplastin time
CT: Computed tomography
LMWH: Low molecular weight heparin
